# Supplementary material for: The Health Economic Evaluation of Bariatric Surgery Versus a Community Weight Management Intervention Analysis from the Idiopathic Intracranial Hypertension Weight Trial (IIH:WT)
Source: Life (Basel). 2021 Apr 30;11(5):409. doi: 10.3390/life11050409 (PMC8146846; doi:10.3390/life11050409)
Supplement: Supplementary file 1 [file life-11-00409-s001.zip › life-1179581-supplementary xml.pdf]

Supplementary Materials:

The Health Economic Evaluation of Bariatric Surgery Versus a Community Weight Management Intervention Analysis from the Idiopathic Intracranial Hypertension Weight Trial (IIH:WT)

Supplementary Table S1. Unit costs (GBP, 2017–18 prices)

Resource Use Item	Unit Cost, GBP	Source
	Interventions	
	Surgery	
Gastric band	5078	
Sleeve	1058	2017/18 Reference Costs
Bypass	5809	
Diet programme (3 months)	48.5	2017/18 Reference Costs
	Primary care	
GP (per consultation)	38	Unit costs of health and social care, 2018
Practice Nurse (per consultation)	10.9	Unit costs of health and social care, 2018
	Secondary care-outpatient	
A&E	148.00	2017/18 Reference Costs
IIH consultation (multi-professional, face-to-face)	225.00	2017/18 Reference Costs
Ophthalmology	139.00	2017/18 Reference Costs
Neurology	178.9	2017/18 Reference Costs
Dietitian	32.3	2017/18 Reference Costs
Adjustment gastric band (post-surgery)	937	2017/18 Reference Costs
Lumbar puncture		2017/18 Reference Costs
	Secondary care-inpatient	
Non-elective spell tariff (trim point = 11)	1323	2017/18 Reference Costs
Tariff adjustment (for extra day in hospital beyond trim point)	240	2017/18 Reference Costs
**Drug name, dose (size of package).**	**Cost per package** (GBP)	
Acetazolamide, 250 mg (110)	16.9	BNF 2018
Acetazolamide Modified release, 250 mg (30)	16.7	BNF 2018
Topiramate 25 mg (60)	1	BNF 2018
Topiramate 50 mg (60)	1.4	BNF 2018
Topiramate 100 mg (60)	2.3	BNF 2018
Amiloride 5 mg (28)	3.2	BNF 2018
Bendroflumethiazide 2.5 mg (28)	0.3	BNF 2018
Bendroflumethiazide 5 mg (28)	0.3	BNF 2018
Furosemide 20 mg (28)	0.4	BNF 2018
Spironolactone 25 mg (28)	1	BNF 2018

Co-amilofruse 2.5 mg/20 mg (28)	5.6	BNF 2018
Co-amilofruse 5 mg/40 mg (28)	7.1	BNF 2018
Co-amilofruse 10 mg/80 mg (28)	15.5	BNF 2018
Atenolol 25 mg (28)	0.4	BNF 2018
Atenolol 50 mg (28)	0.4	BNF 2018
Atenolol 100 mg (28)	0.5	BNF 2018
Candesartan 2 mg (28)	9.9	BNF 2018
Labetalol hydrochloride 5 mg/mL (x 5)	59.2	BNF 2018
Amlodipine 5 mg (28)	1.4	BNF 2018
Ramipril 2.5 mg (28)	0.6	BNF 2018
Amitriptyline 10 mg (28)	1	BNF 2018
Gabapentin 600 mg (100)	6.5	BNF 2018
Gabapentin 800 mg (100)	27.4	BNF 2018
Dosulepin 75 mg (28)	1.2	BNF 2018
Dosulepin 25 mg (28)	0.9	BNF 2018
Propranolol 10 mg (28)	0.9	BNF 2018
Nortriptyline 25 mg (100)	11.6	BNF 2018
Zonisamide 50 mg (56)	34.3	BNF 2018
Mirtazapine 30 mg (28)	1	BNF 2018

Supplementary Table S2. Data completeness.

	**Community weight Management Intervention**			**Bariatric Surgery Pathway**		
	Complete	Incomplete (imputed)	Total	Complete	Incomplete (imputed)	Total
		**12 months**				
Intracranial pressure	25	8	33	29	4	33
GP Visits	27	6	33	29	4	33
Nurse Visits	24	9	33	27	6	33
Number of outpatient visits	33	0	33	33	0	33
Number of days in hospital	33	0	33	33	0	33
		**24 months**				
Intracranial pressure	18	15	33	22	11	33
GP Visits	23	10	33	23	10	33
Nurse Visits	19	14	33	23	10	33
Number of outpatient visits	32	1	33	33	0	33
Number of days in hospital	31	2	33	33	0	33
